# Supplementary material for: Computational epitope map of SARS-CoV-2 spike protein
Source: PLoS Comput Biol. 2021 Apr 1;17(4):e1008790. doi: 10.1371/journal.pcbi.1008790 (PMC8016105; doi:10.1371/journal.pcbi.1008790)
Supplement: S1 Text — (PDF) [file pcbi.1008790.s001.pdf]

Supplementary Information: Computational epitope map of SARS-CoV-2 spike protein

Mateusz Sikora[1,◑], Sören von Bülow[1,◑] Florian E. C. Blanc[1,◑] Michael Gecht[1,◑] Roberto Covino[1,2◑] Gerhard Hummer[1,3,*]

1 Department of Theoretical Biophysics, Max Planck Institute of Biophysics, Frankfurt am Main, Germany. **2** Frankfurt Institute for Advanced Studies, Frankfurt am Main, Germany. **3** Institute of Biophysics, Goethe University Frankfurt, Frankfurt am Main, Germany.

◑These authors contributed equally to this work.
* gerhard.hummer@biophys.mpg.de (G.H.)

S1 Text: Supplementary Methods

Full-length molecular model of SARS-CoV-2 S glycoprotein. The modeling procedure of the full-length SARS-CoV-2 S glycoprotein is outlined in S6 Fig. We based our model of the SARS-CoV-2 S1/S2 S domain on a recently determined structure (PDB ID: 6VSB [1]). We added missing loops by using MODELLER [2]. We modeled the stalk connecting the S head to the membrane as two distinct coiled coils (CCs, henceforth denoted CC1 and HR2) based on CC predictions [3, 4]. CC1 and HR2 at positions 1138-1158 and 1167-1204 are predicted with low and high confidence, respectively. However, since the N-terminal ends of the three helices in CC1 have been resolved in the experimental structures [1, 5], we modeled both segments as trimeric CCs with CCBuilder [6], using the heptad repeat register prediction of [3] and generously extending all termini by several residues to prevent destabilization of the CCs from solvation effects at the termini. Thus, the first model of CC1 comprised residues 1137-1163, while HR2 comprised residues 1161-1214. We then performed 1 µs-long MD simulations of the solvated CC1 and HR2 models individually with procedures and parameter settings as described below. In CC1 and HR2, residues 1138-1158 and 1167-1204 retained stable CC structures, respectively. The CC structures of snapshots at 390 ns (CC1) and 166 ns (HR2) were integrated into a model of full-length SARS-CoV-2 S.

Glycosylation of S ectodomain and connector domain. There are 22 N-glycosylation sequons present on the surface of S, all of which have been confirmed recently by mass spectrometry of a recombinant protein [7]. Distinct glycan types are preferred on various sequons, with the majority being oligomannose, followed by sialylated and fucosylated complex glycans and a minority of the hybrid type. Here we selected the most abundant species at each site, as shown in S1 Fig. All fucose residues were linked in α-1,3 position and sialic acid in α-2,3. Consistent with the low glycan occupancy in the structure *in situ* [8], O-glycosylation in positions 323 and 325 was not included. Contrary to some observations [9], the complete glycosylation pattern including heavy glycosylation of the stalk seems to reflect better the situation *in situ* [8].

Modeling of the transmembrane domain. Lacking a structure for the S transmembrane domain (TMD), we used a hierarchical procedure to model the TMD trimer. Secondary structure predictions revealed that the TMD is likely to be formed of two helical stretches with a long transmembrane helix (residues 1212-1237), followed by a shorter C-terminal helix (residues 1242-1249) with features of an amphipathic helix. The remaining 24 C-terminal residues were predicted as disordered. We hypothesized that the C-terminal helix extends to K1255 and encompasses all cysteine residues, leaving a total of 18 disordered residues at the C-terminus.

We modelled the TMD helical core (residues 1208-1237) as three perfect α-helices in a tripod arrangement. We palmitoylated all cysteines, inserted the trimer into a lipid bilayer (see below and Table C, and relaxed the system using molecular dynamics (MD; see parameters below) for 1 µs, to properly equilibrate the relative orientation of the protomers.

Separately, we built an L-shaped TMD monomer model by appending the C-terminal helix (residues 1243-1265, modeled as an ideal α-helix) to the TMD core helix (residues 1208-1237). The C-terminal helix was oriented such that all cysteines pointed into the hydrophobic core of the membrane. The five residues connecting the TMD and C-terminal helix, as well as the 18 C-terminal residues were modeled as unstructured loops using MODELLER [2]. All cysteines were palmitoylated and the monomer was inserted into a lipid bilayer, then relaxed by molecular dynamics for 1 µs for proper positioning of the C-terminal helix with respect to the lipid head groups.

Finally, a TMD trimer model was obtained by structurally fitting the relaxed L-shaped monomer onto the relaxed transmembrane trimer. In two out of three monomers, this resulted in an outward-pointing, clash-free C-terminal helix. In the third monomer, the C-terminal helix was manually rotated around the z-axis to relieve clashes.

Assembly of full-length S model. A full-length model of S was built by manually matching the separate structural domains using PyMOL [10], and then building missing connecting residues as unstructured linkers with MODELLER [2].

Comparison with the model by Casalino et al Our modelling decisions are similar to those by Casalino et al. [11] except on the following points:

1. We modelled the spike head with one chain open and two chains closed (6VSB cryo-EM structure). In addition to this configuration, Casalino et al. also considered a spike model with all three chains closed (6VXX cryo-EM structure).

2. We manually modelled the C-terminal domain as an alpha-helix flanked by disordered linkers, which we relaxed by MD. By contrast, Casalino et al. used the I-TASSER structural prediction server. Interestingly, they retained a model with an alpha-helix similar to ours.

3. We decided to palmitoylate all cysteines in the TMD and C-terminal domains. Casalino et al. palmitoylated only cysteines 1240 and 1241.

4. Similar to Casalino et al., we followed the N-glycosylation determined by Wanatabe et al., with essentially the same glycan types (including large tetra-antennary glycans on the stalk) and small differences in the details of sialylation and/or fucosylation. However, we did not include any O-glycans. In addition, Casalino et al. considered a system with N165 and N234 mutated to alanine. We instead performed a large-scale resampling of the simulations to understand the role of glycan size and composition on shielding.

Overall, most differences relate to secondary structure assignment in the stalk and global structural modelling of the TMD/C-terminal region including the palmitoylation pattern. As high-resolution structural data of these regions are currently lacking, the disparity of modelling decisions between independent studies may actually be useful in exploring plausible solutions. Also, we note that possible modelling errors of the TMD/C-terminal regions are expected to have very little bearing on the protruding, solvent-exposed regions of the spike which are most relevant for the present epitope search.

Membrane lipid composition. Coronaviruses like MERS-CoV and SARS-CoV are assembled in the endoplasmic reticulum (ER) [12]. We therefore modeled the viral envelope with an ER-like composition [13] as detailed in Table C. The transmembrane domain structures described above were inserted into the ER-like membrane using CHARMM-GUI [14–18].

Molecular dynamics simulations. Molecular dynamics simulations were performed with GROMACS 2019.6 [19], using the CHARMM36m protein and glycan force field [20–22], in combination with the TIP3P water model [23]. Ion parameters were those by Luo and Roux [24].

After energy minimization using the steepest descent algorithm for 55 000 steps, the system was equilibrated in the NVT ensemble for 375 ps with a time step of 1 fs, followed by 1500 ps with a time step of 2 fs. In the equilibration runs, the Berendsen thermostat [25] was used for temperature coupling, with the coupling constant $\tau = 1$ ps. After 250 ps, we used the Parrinello-Rahman barostat [26] to apply semiisotropic pressure coupling, using $\tau = 5$ ps and compressibility $4.5 \times 10^{-5}\,\mathrm{bar}^{-1}$. LINCS constraints [27] were applied to all bonds involving hydrogen atoms, allowing us to use a 2 fs integration timestep for equilibration. During equilibration, restraints on positions and dihedrals were gradually decreased from $1000\,\mathrm{kJ\,mol^{-1}\,nm^{-2}}$ to 0.

Due to the large system size, we adopted specific strategies to enhance the simulation speed during production. We used an integration timestep of 4 fs. All hydrogen masses were doubled, corresponding to deuterium, to avoid instabilities from high frequency vibrations. Cutoffs for non-bonded interactions were set to 1 nm. In addition, temperature control was switched to the Velocity-Rescale thermostat [28]. We used MDBenchmark to perform scaling studies and determine the optimal hardware configuration and run settings (MPI ranks/OpenMP threads) [29].

Rigid body docking. We probed the steric accessibility for antibody binding using rigid body docking. The Fab of antibody CR3022 (PDB ID: 6W41 [30]) was used for a coarse-grained rigid body Monte Carlo (MC) docking analysis according to the simulation procedure described in [31]. Backbone C_α atoms of single S were recorded every 10 ns of the MD simulation of four S embedded in the membrane. Each snapshot was centered in a 24.5 nm × 24.5 nm × 36 nm orthorhombic simulation box. The Fab was subjected to 2×10^5 translation and rotation MC moves, recorded every 20 moves.

In a first step, we probed the steric accessibility of the protein surface without glycans using rigid body MC simulations at high temperature ($T = 10\,000$ K). Contacts between the complementarity-determining region of the Fab (heavy chain residues 31-35, 50-65, 95-102; and light chain residues 24-34, 50-56, 89-97) and S were then counted based on a distance criterion of twice the sum of van der Waals (vdW) radii of the amino acids involved in the contact (with radius definitions following [31]). MC simulation snapshots with steric clashes between Fab and lipids were removed from the analysis.

In a second step, we assessed the influence of glycans on the steric surface coverage by excluding all snapshots in which the Fab clashed with glycans. Every sugar residue

of a glycan was represented by a pseudoparticle positioned at the residue center of mass. The effective vdW radius of this sugar bead was estimated from the sugar residue radius of gyration and found to be roughly equal to the vdW radius of an alanine residue, as defined in [31]. A distance cutoff of the sum of Fab residue vdW radius and glycan ($\approx$ alanine) vdW radius was used to determine clashes.

We further tested the accessibility of the protein surface in the absence of complex glycans, i.e. with only oligo-mannose glycosylation present. To this end, for each snapshot and each mannose-5 site we extracted glycan positions along with corresponding asparagine and two flanking residues, in this way creating a library of possible conformers. Next, for each glycosylation site, each spike protein and each frame of the trajectory we picked a conformer at random, rigid-body aligned the protein backbone atoms onto the site and repeated the procedure until no clashes with corresponding spike protein within 0.75 Å were observed. We then repeated the docking accessibility analysis on this resampled glycan shield.

Finally, atoms of neighboring S with a minimum distance of $\leq$3 nm were included in the analysis to assess the effect of protein crowding on the Fab accessibility to the protein, analogous to the procedure in the ray accessibility analysis.

Relative accessibility reduction due to the glycan shield. We quantified the glycan coverage by comparing global accessibility (to rays or to the rigid, coarse-grained Fab) of the S surface with various glycosylation patterns and without glycans. First, the global accessibility was computed as the sum over all residues of the numbers of hits for a given probing method and glycosylation pattern. Then, we considered the ratio of global accessibility with glycans over global accessibility without glycans. Finally, the relative accessibility reduction due to glycan coverage was taken as the complementary of this global accessibility ratio
(relative accessibility reduction = 1 − global accessibility ratio).

Contact map calculation To calculate an inter-S contact map, we used a simplified representation with only C_α atoms of the protein and ring oxygen atoms O5 of glycans (O6 atom in the sialic acid). For each simulation frame, we defined a contact if the periodic boundary-corrected distance between residues/saccharides belonging to two distinct S was below 8.5 Å. Finally, we averaged the total contact count over chains of S (3), number of S (4) and number of snapshots (250).

**Observable**	x_0	x_1
Sequence conservation	0.9985	1.0
BepiPred score	0.35	0.55
Local rigidity $\left[\text{Å}^{-1}\right]$	0.2	2.5
Ray hits	1000	3000
Rigid body docking hits	5	1500
Consensus score	0	0.2

Table A. Mapping of an individual scores x to the interval $[0, 1]$. $x \leq x_0$ is mapped to 0, $x \geq x_1$ is mapped to 1, and linear interpolation is used in between.

**System**	**Rays**	**Docking**
Full glycosylation	34%	80%
Full glycosylation and crowding	39%	86%
Mannose-5-like glycosylation (Man5)	NA	75%

Table B. Relative reduction in surface accessibility due to glycan coverage.

**Lipid**	**Full name**	**%**
DOPC	1,2-dioleoyl-glycero-3-phosphocholine	25
POPC	1-palmitoyl-2-oleoyl-glycero-3-phosphocholine	25
POPE	1-palmitoyl-2-oleoyl-glycero-3-phosphoethanolamine	20
POPI	1-palmitoyl-2-oleoyl-glycero-3-phosphoinositol	15
POPS	1-palmitoyl-2-oleoyl-sn-glycero-3-phospho-L-serine	5
CER160	N-palmitoyl-D-erythro-sphingosine	5
CHOL	Cholesterol	5

Table C. ER-like membrane composition used in the MD simulations.

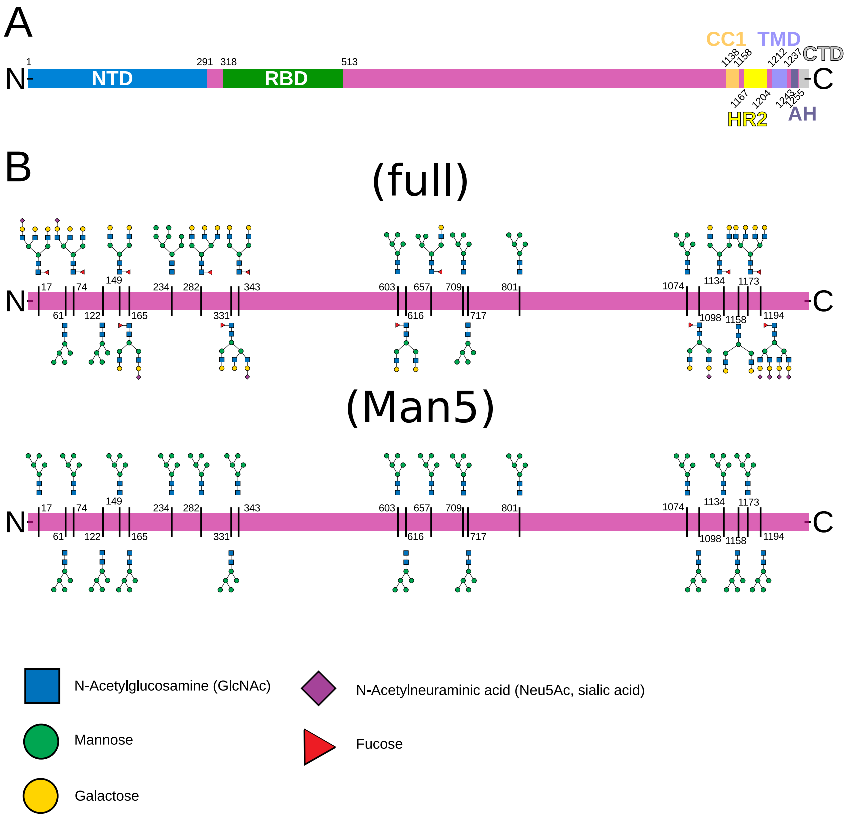

Fig S1. Spike domains and glycosylation. (A) Domains of S. (B) Glycosylation pattern of S. Sequons are indicated with the respective glycans in a schematic representation for a fully glycosylated system ("full") and for resampled simulations containing only mannose-5 ("Man5").

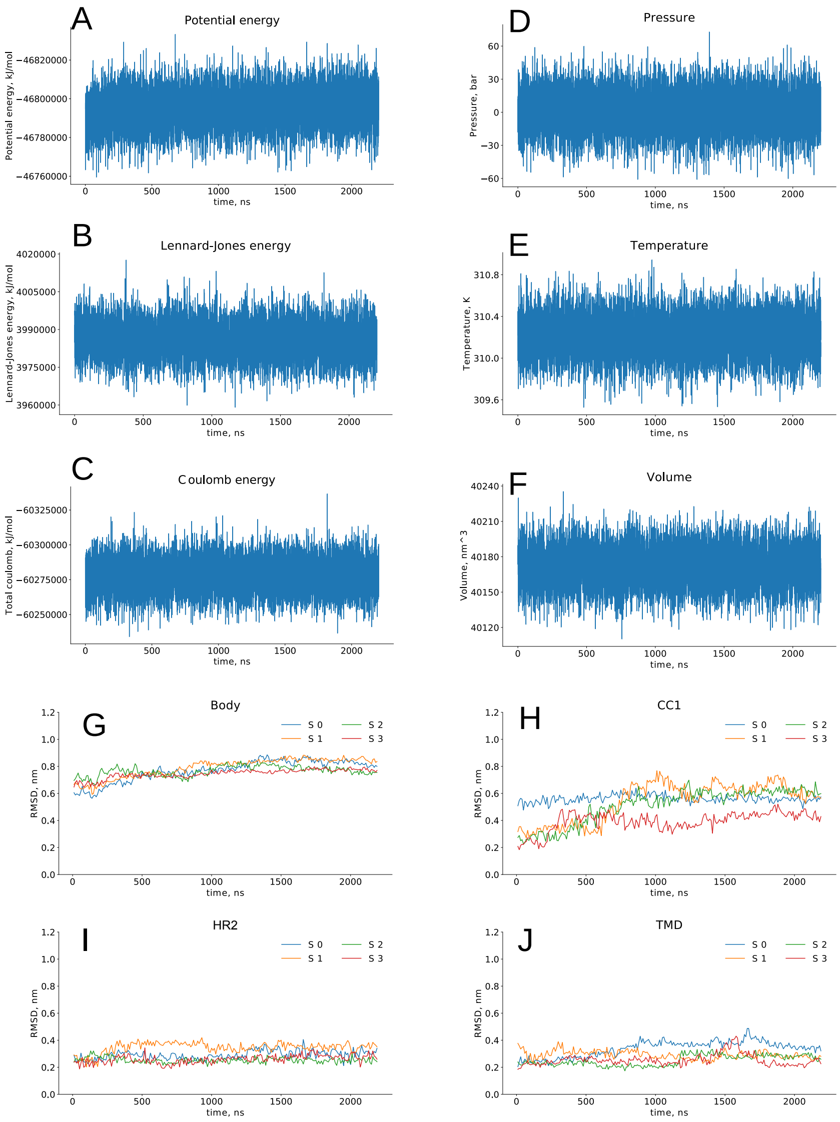

Fig S2. Time series of various key parameters monitored during the simulation. (A) Total potential energy, (B) Lennard-Jones energy, (C) Coulomb energy, (D-F) temperature, pressure, and volume of the simulation box. (G-J) Root-mean-square deviation (RMSD) over the course of the simulation, calculated for C_α carbons of the S body, CC1, HR2, and TMD, with respect to a reference configuration obtained after 300 ns of equilibration. Values for four spike proteins are shown with distinct colors.

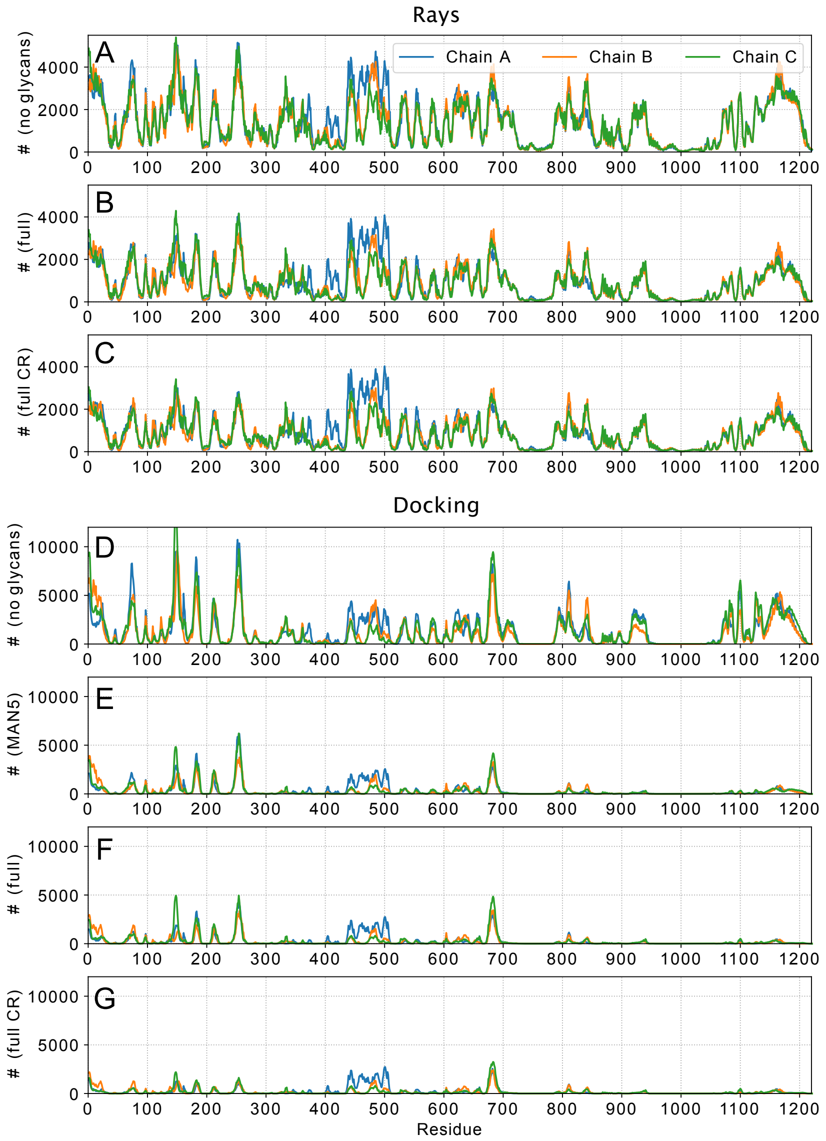

Fig S3. Impact of the glycosylation pattern on ray (A-C) and docking (D-G) accessibility. (A-C) Number of ray hits without glycans ("no glycans"), with full glycans ("full", S1B Fig), and with full glycans and S protein crowding ("full CR"). (D-G) Monte Carlo rigid body docking hits without glycans ("no glycans"), with Man5 glycans ("Man5", S1B Fig) and with full glycans ("full"), as well as with full glycans and S protein crowding ("full CR").

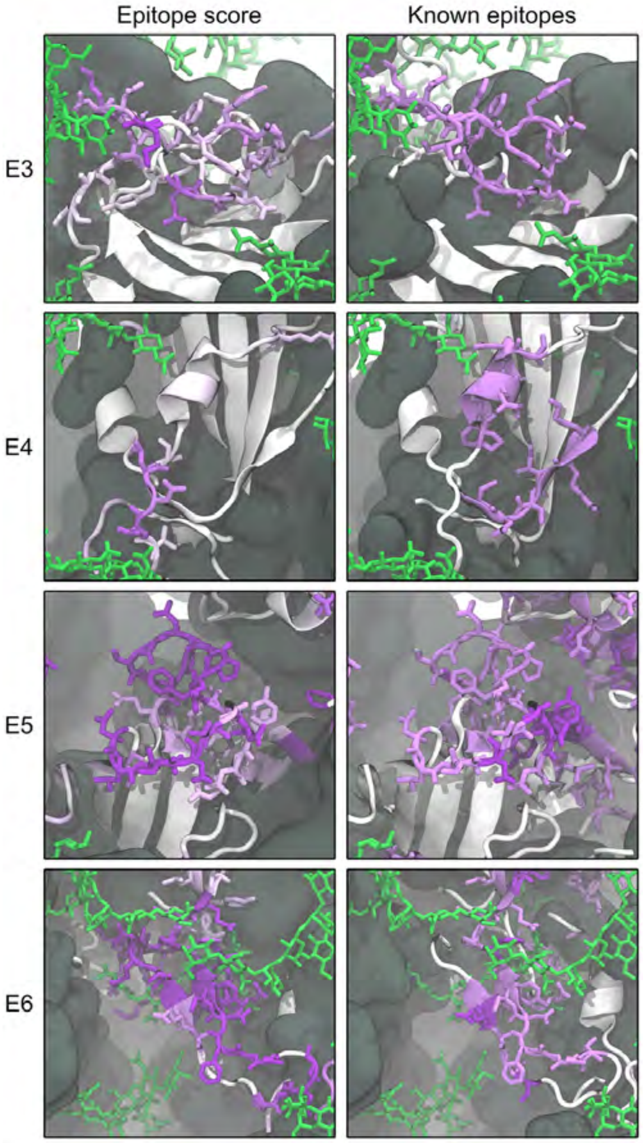

Fig S4. Comparison of the epitope candidates E3–E6 with previously characterized epitopes. Glycans are shown in green licorice representation. Left panels: Epitope candidates shown in cartoon representation with purple color intensity indicating epitope consensus scores. Residues with epitope consensus score >0.1 are shown in licorice representation. Right panels: Epitopes described in previous works shown in cartoon and licorice representation, with higher purple color intensity indicating reported binding to multiple distinct antibodies.

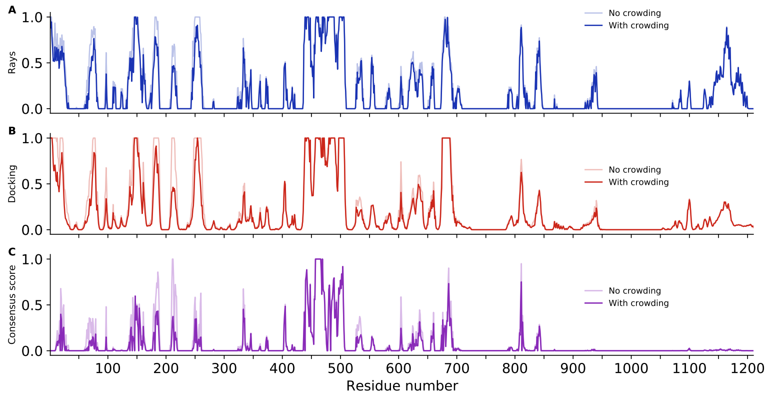

Fig S5. Effect of crowding on accessibility and epitope score. (A) Ray, (B) docking and (C) consensus scores with (thick line) and without crowding being taken into account.

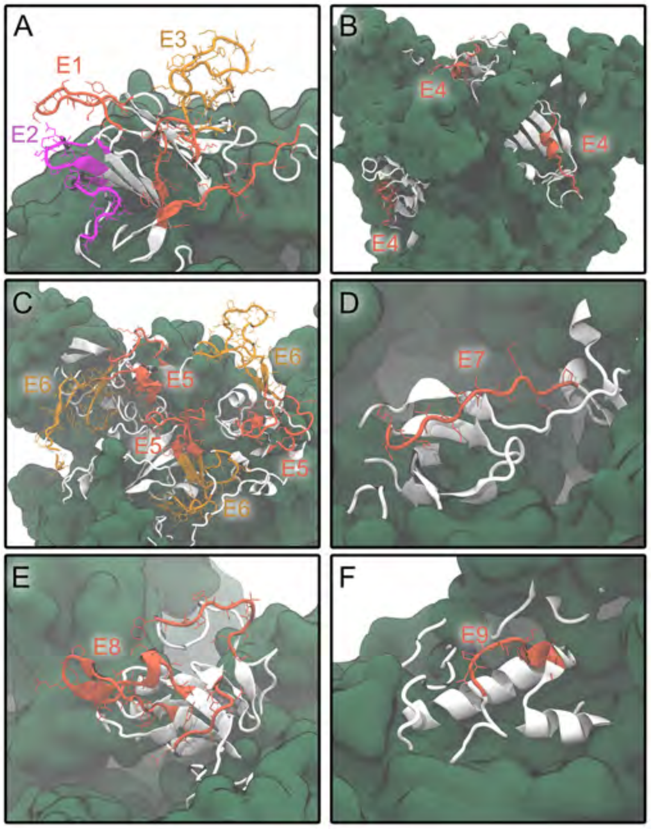

Fig S6. Location and structural features of the epitope candidates E1–E9 on the S surface. Epitope candidates are shown in red, orange and purple cartoon and licorice representation. Neighboring residues are shown in grey cartoon representation.

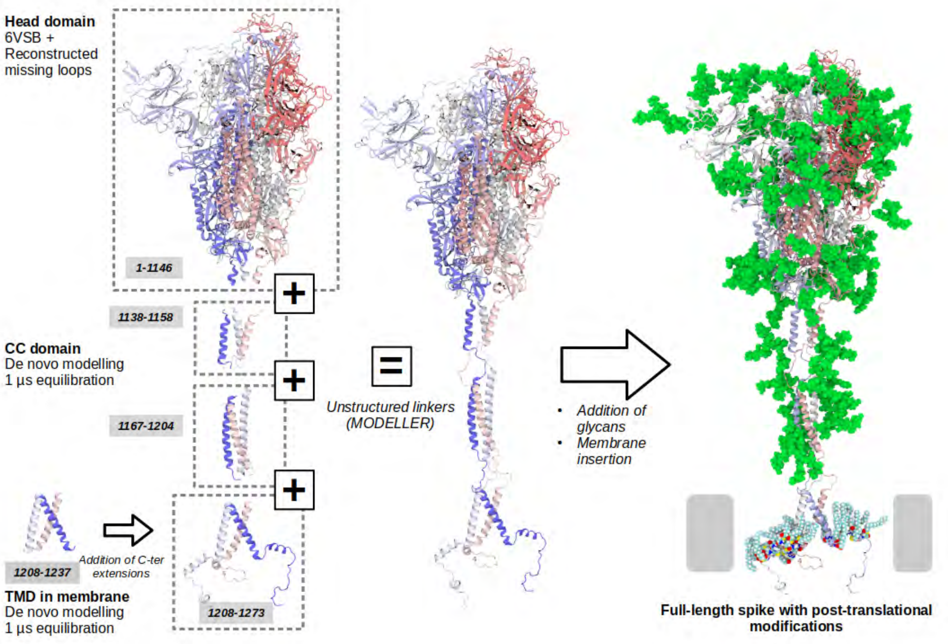

Fig S7. Schematic illustration of the strategy used to obtain an atomistic model of the full-length S protein. For clarity, we do not show the solvent and membrane.

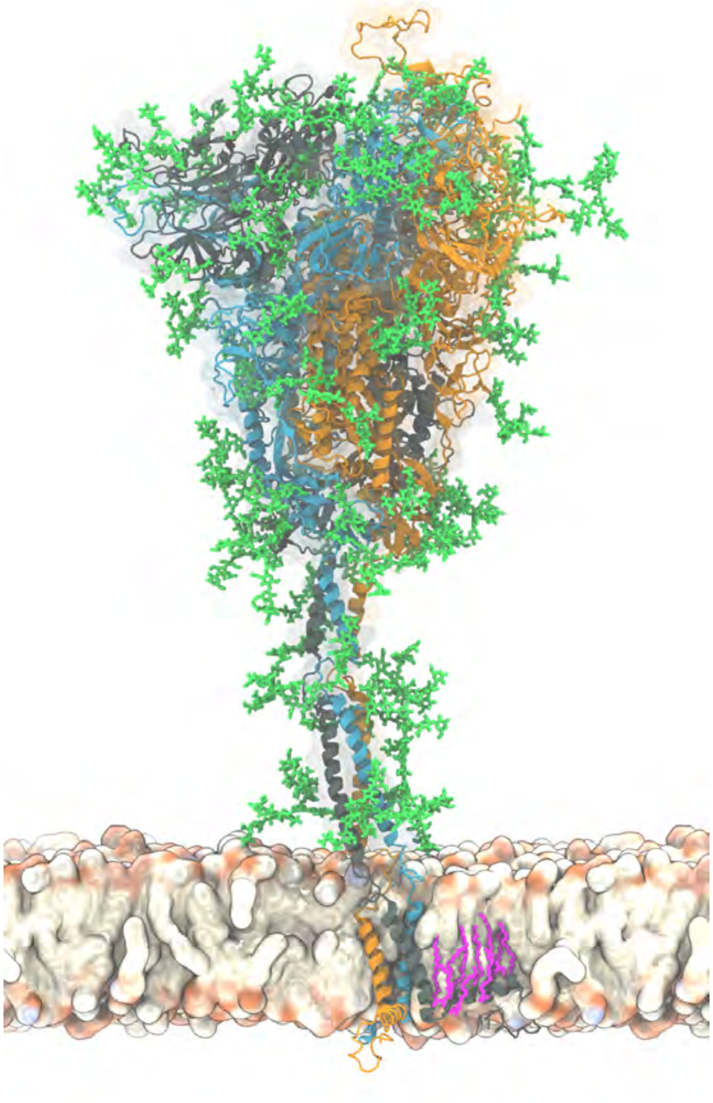

Fig S8. Atomistic model of the full-length membrane-embedded S protein shown in cartoon representation. The chains are differentiated by color. Palmitoylated cysteine residues are shown in pink licorice (only one chain shown for clarity). Glycans are shown in green licorice representation. We show a section of the membrane to highlight the transmembrane domain of S.

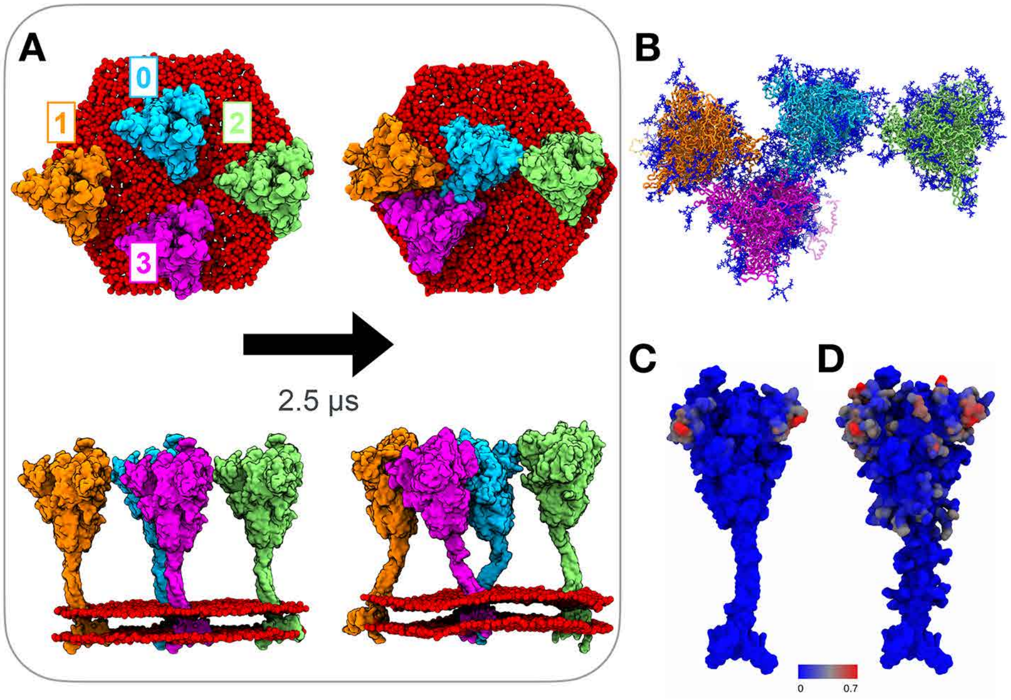

Fig S9. Spike-spike interactions and bending during MD simulation. (A) Snapshots of the 4-spike system from above (top row) and in side-view (bottom row) at the beginning (left) and end (right) of the MD trajectory. While the transmembrane regions move relatively little, spike heads form spike-spike interactions because of significant bending at the "knee" (CC1 - CC2 joint). These interactions persist on the simulation timescale. (B) Visualization of the glycans in the final configuration (blue sticks). Glycans mediate spike-spike contacts. (C and D) Maps of time-averaged spike-spike contact probability mediated by amino-acids (C) or amino-acids and glycans (D) from the MD trajectory (color bar: contact probability). Interactions are located exclusively on lateral faces of the spike head.

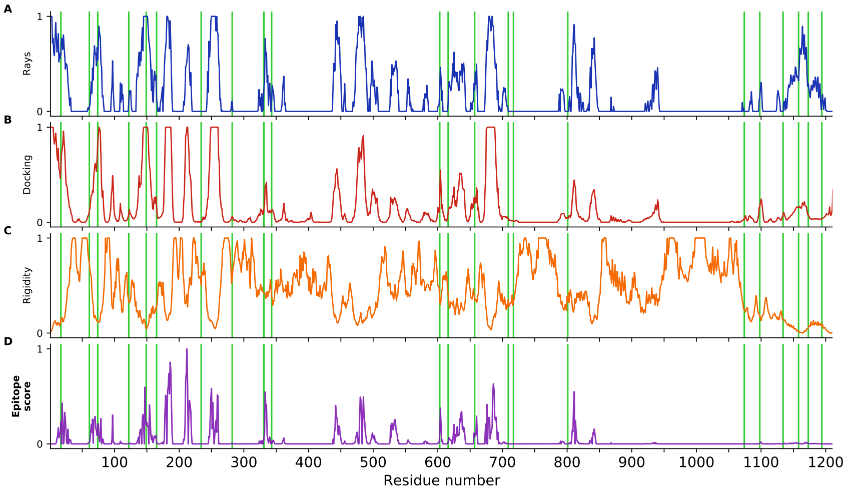

Fig S10. Consensus score analysis of "closed" spike. (A, B) Accessibility, (C) rigidity and (D) consensus score calculated taking only into account the chains with down RBDs.

Movie S1. Atomistic molecular dynamics simulation trajectory of four S proteins embedded in a membrane. The proteins and lipids are shown in surface representation. Glycans are represented by green van der Waals beads. Water and ions are omitted for clarity. 600 ns simulation time shown.

References

1. Walls AC, Park YJ, Tortorici MA, Wall A, McGuire AT, Veesler D. Structure, Function, and Antigenicity of the SARS-CoV-2 Spike Glycoprotein. Cell. 2020;181(2):281–292.e6. doi:10.1016/j.cell.2020.02.058.
2. Eswar N, Webb B, Marti-Renom MA, Madhusudhan MS, Eramian D, Shen My, et al. Comparative Protein Structure Modeling Using Modeller. Current Protocols in Bioinformatics. 2006;15(1):5.6.1–5.6.30. doi:10.1002/0471250953.bi0506s15.
3. Lupas A, Van Dyke M, Stock J. Predicting coiled coils from protein sequences. Science. 1991;252(5009):1162–1164.
4. Vincent TL, Green PJ, Woolfson DN. LOGICOIL–multi-state prediction of coiled-coil oligomeric state. Bioinformatics. 2013;29(1):69–76. doi:10.1093/bioinformatics/bts648.
5. Wrapp D, Wang N, Corbett KS, Goldsmith JA, Hsieh CL, Abiona O, et al. Cryo-EM structure of the 2019-nCoV spike in the prefusion conformation. Science. 2020;367(6483):1260–1263. doi:10.1126/science.abb2507.
6. Wood CW, Woolfson DN. CCBuilder 2.0: powerful and accessible coiled-coil modeling. Protein Science. 2018;27(1):103–111. doi:10.1002/pro.3279.
7. Watanabe Y, Allen JD, Wrapp D, McLellan JS, Crispin M. Site-specific glycan analysis of the SARS-CoV-2 spike. Science. 2020;doi:10.1126/science.abb9983.
8. Turoňová B, Sikora M, Schürmann C, Hagen WJH, Welsch S, Blanc FEC, et al. In situ structural analysis of SARS-CoV-2 spike reveals flexibility mediated by three hinges. bioRxiv. 2020;doi:10.1101/2020.06.26.173476.
9. Shajahan A, Supekar NT, Gleinich AS, Azadi P. Deducing the N- and O-glycosylation profile of the spike protein of novel coronavirus SARS-CoV-2. Glycobiology. 2020;doi:10.1093/glycob/cwaa042.
10. The PyMOL Molecular Graphics System, Schrödinger, LLC.;. Available from: `https://pymol.org/`.
11. Casalino L, Gaieb Z, Goldsmith JA, Hjorth CK, Dommer AC, Harbison AM, et al. Beyond Shielding: The Roles of Glycans in the SARS-CoV-2 Spike Protein. ACS Central Science. 2020;6(10):1722–1734. doi:10.1021/acscentsci.0c01056.
12. Masters PS. The molecular biology of coronaviruses. In: Advances in Virus Research. vol. 66; 2006. p. 193–292. Available from: `https://www.sciencedirect.com/science/article/pii/S0065352706660053`.
13. Jacquemyn J, Cascalho A, Goodchild RE. The ins and outs of endoplasmic reticulum-controlled lipid biosynthesis. EMBO Reports. 2017;18(11):1905–1921. doi:10.15252/embr.201643426.
14. Jo S, Kim T, Im W. Automated Builder and Database of Protein/Membrane Complexes for Molecular Dynamics Simulations. PLOS ONE. 2007;2(9):e880. doi:10.1371/journal.pone.0000880.
15. Jo S, Kim T, Iyer VG, Im W. CHARMM-GUI: a web-based graphical user interface for CHARMM. Journal of Computational Chemistry. 2008;29(11):1859–1865. doi:10.1002/jcc.20945.

16. Jo S, Lim JB, Klauda JB, Im W. CHARMM-GUI membrane builder for mixed bilayers and its application to yeast membranes. Biophysical Journal. 2009;97(1):50–58. doi:10.1016/j.bpj.2009.04.013.

17. Wu EL, Cheng X, Jo S, Rui H, Song KC, DÃ¡vila-Contreras EM, et al. CHARMM-GUI Membrane Builder toward realistic biological membrane simulations. Journal of Computational Chemistry. 2014;35(27):1997–2004. doi:10.1002/jcc.23702.

18. Lee J, Cheng X, Swails JM, Yeom MS, Eastman PK, Lemkul JA, et al. CHARMM-GUI Input Generator for NAMD, GROMACS, AMBER, OpenMM, and CHARMM/OpenMM Simulations Using the CHARMM36 Additive Force Field. Journal of Chemical Theory and Computation. 2016;12(1):405–413. doi:10.1021/acs.jctc.5b00935.

19. Abraham MJ, Murtola T, Schulz R, Páll S, Smith JC, Hess B, et al. GROMACS: high performance molecular simulations through multi-level parallelism from laptops to supercomputers. SoftwareX. 2015;1-2:19–25. doi:10.1016/j.softx.2015.06.001.

20. Huang J, Rauscher S, Nawrocki G, Ran T, Feig M, de Groot BL, et al. CHARMM36m: an improved force field for folded and intrinsically disordered proteins. Nature Methods. 2017;14(1):71–73. doi:10.1038/nmeth.4067.

21. Guvench O, Hatcher E, Venable RM, Pastor RW, MacKerell AD. CHARMM additive all-atom force field for glycosidic linkages between hexopyranoses. Journal of Chemical Theory and Computation. 2009;5(9):2353–2370. doi:10.1021/ct900242e.

22. Park SJ, Lee J, Qi Y, Kern NR, Lee HS, Jo S, et al. CHARMM-GUI glycan modeler for modeling and simulation of carbohydrates and glycoconjugates. Glycobiology. 2019;29(4):320–331. doi:10.1093/glycob/cwz003.

23. Jorgensen WL, Chandrasekhar J, Madura JD, Impey RW, Klein ML. Comparison of simple potential functions for simulating liquid water. The Journal of Chemical Physics. 1983;79(2):926–935. doi:10.1063/1.445869.

24. Luo Y, Roux B. Simulation of osmotic pressure in concentrated aqueous salt solutions. Journal of Physical Chemistry Letters. 2010;1(1):183–189. doi:10.1021/jz900079w.

25. Berendsen HJC, Postma JPM, van Gunsteren WF, DiNola A, Haak JR. Molecular dynamics with coupling to an external bath. The Journal of Chemical Physics. 1984;81(8):3684–3690. doi:10.1063/1.448118.

26. Parrinello M, Rahman A. Polymorphic transitions in single crystals: a new molecular dynamics method. Journal of Applied Physics. 1981;52(12):7182–7190. doi:10.1063/1.328693.

27. Hess B, Bekker H, Berendsen HJC, Fraaije JGEM. LINCS: A linear constraint solver for molecular simulations. Journal of Computational Chemistry. 1997;18(12):1463–1472. doi:10.1002/(SICI)1096-987X(199709)18:12<1463::AID-JCC4>3.0.CO;2-H.

28. Bussi G, Donadio D, Parrinello M. Canonical sampling through velocity rescaling. The Journal of Chemical Physics. 2007;126(1):014101. doi:10.1063/1.2408420.

29. Gecht M, Siggel M, Linke M, Hummer G, Köfinger J. MDBenchmark: A toolkit to optimize the performance of molecular dynamics simulations. The Journal of Chemical Physics. 2020;153(14):144105. doi:10.1063/5.0019045.

30. Yuan M, Wu NC, Zhu X, Lee CCD, So RTY, Lv H, et al. A highly conserved cryptic epitope in the receptor binding domains of SARS-CoV-2 and SARS-CoV. Science. 2020;368(6491):630–633. doi:10.1126/science.abb7269.

31. Kim YC, Hummer G. Coarse-grained models for simulations of multiprotein complexes: application to ubiquitin binding. Journal of Molecular Biology. 2008;375(5):1416–1433. doi:10.1016/j.jmb.2007.11.063.
