# Supplementary figures and images for: Computational epitope map of SARS-CoV-2 spike protein
Source: PLoS Comput Biol. 2021 Apr 1;17(4):e1008790. doi: 10.1371/journal.pcbi.1008790 (PMC8016105; doi:10.1371/journal.pcbi.1008790)

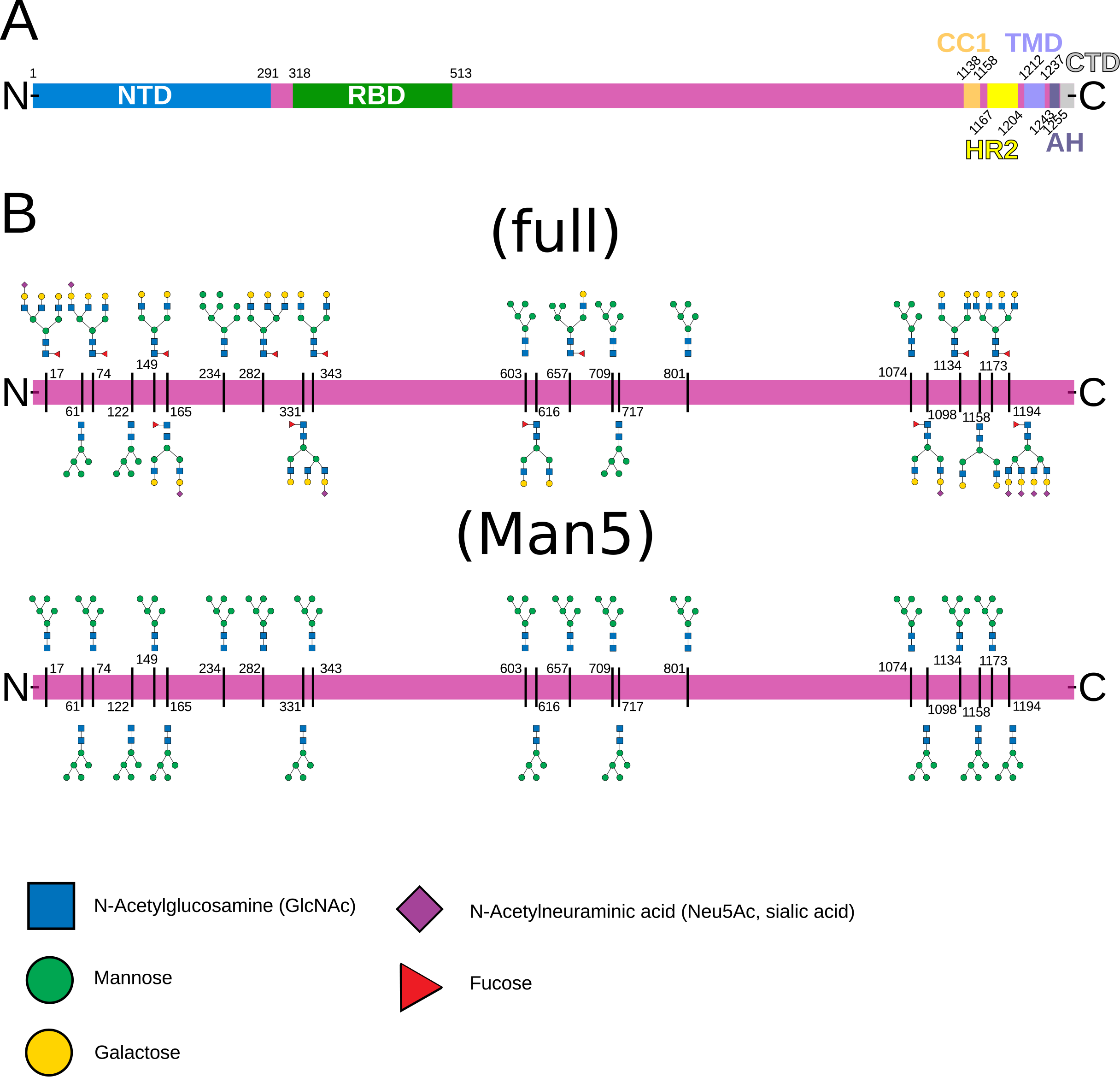

Supplement: S1 Fig — (A) Domains of S. (B) Glycosylation pattern of S. Sequons are indicated with the respective glycans in a schematic representation for a fully glycosylated system (“full”) and for resampled simulations containing only mannose-5 (“Man5”). (TIF) [file pcbi.1008790.s002.tif]

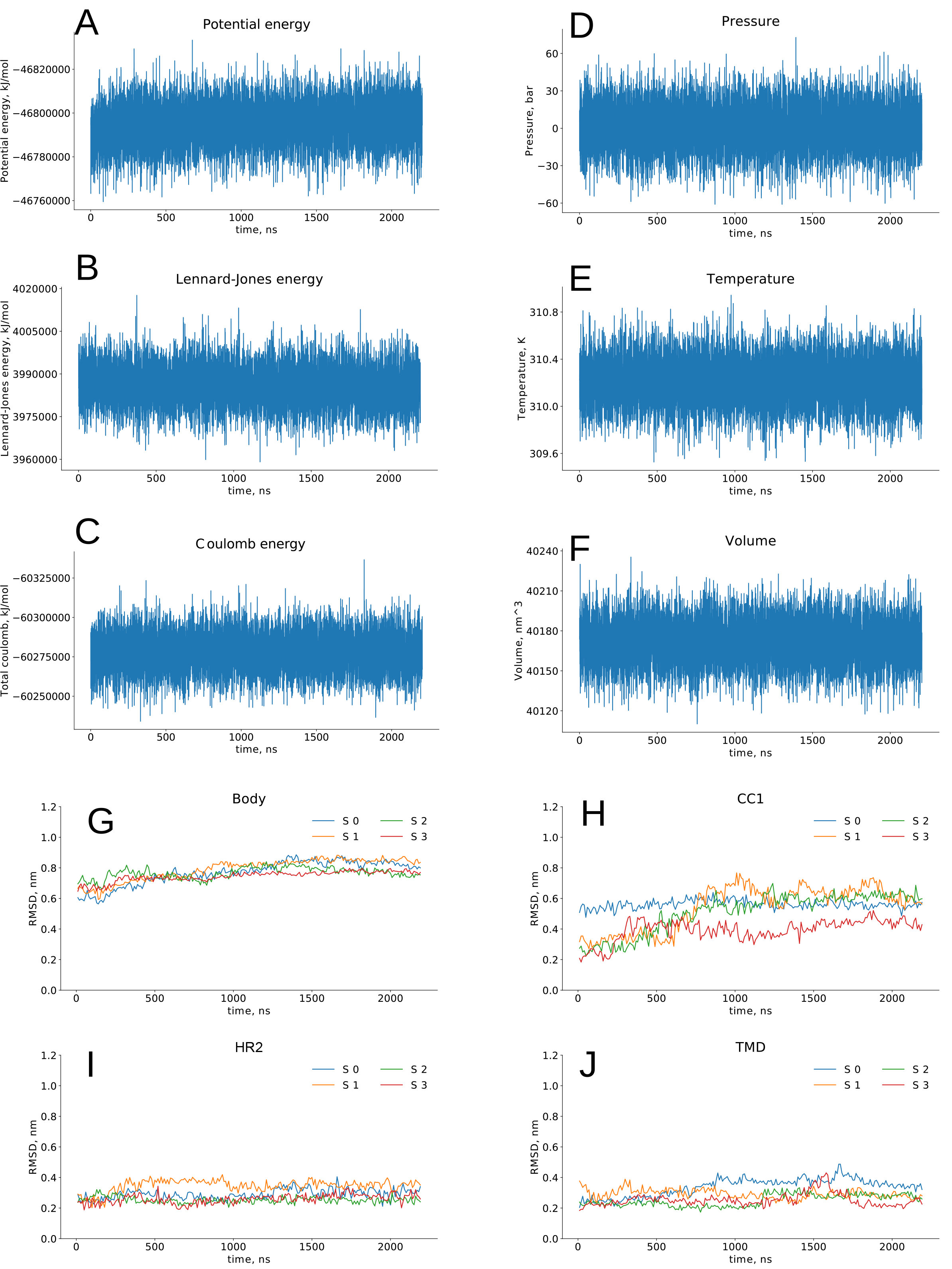

Supplement: S2 Fig — (A) Total potential energy, (B) Lennard-Jones energy, (C) Coulomb energy, (D-F) temperature, pressure, and volume of the simulation box. (G-J) Root-mean-square deviation (RMSD) over the course of the simulation, calculated for Cα carbons of the S body, CC1, HR2, and TMD, with respect to a reference configuration obtained after 300 ns of equilibration. Values for four spike proteins are shown with distinct colors. (TIF) [file pcbi.1008790.s003.tif]

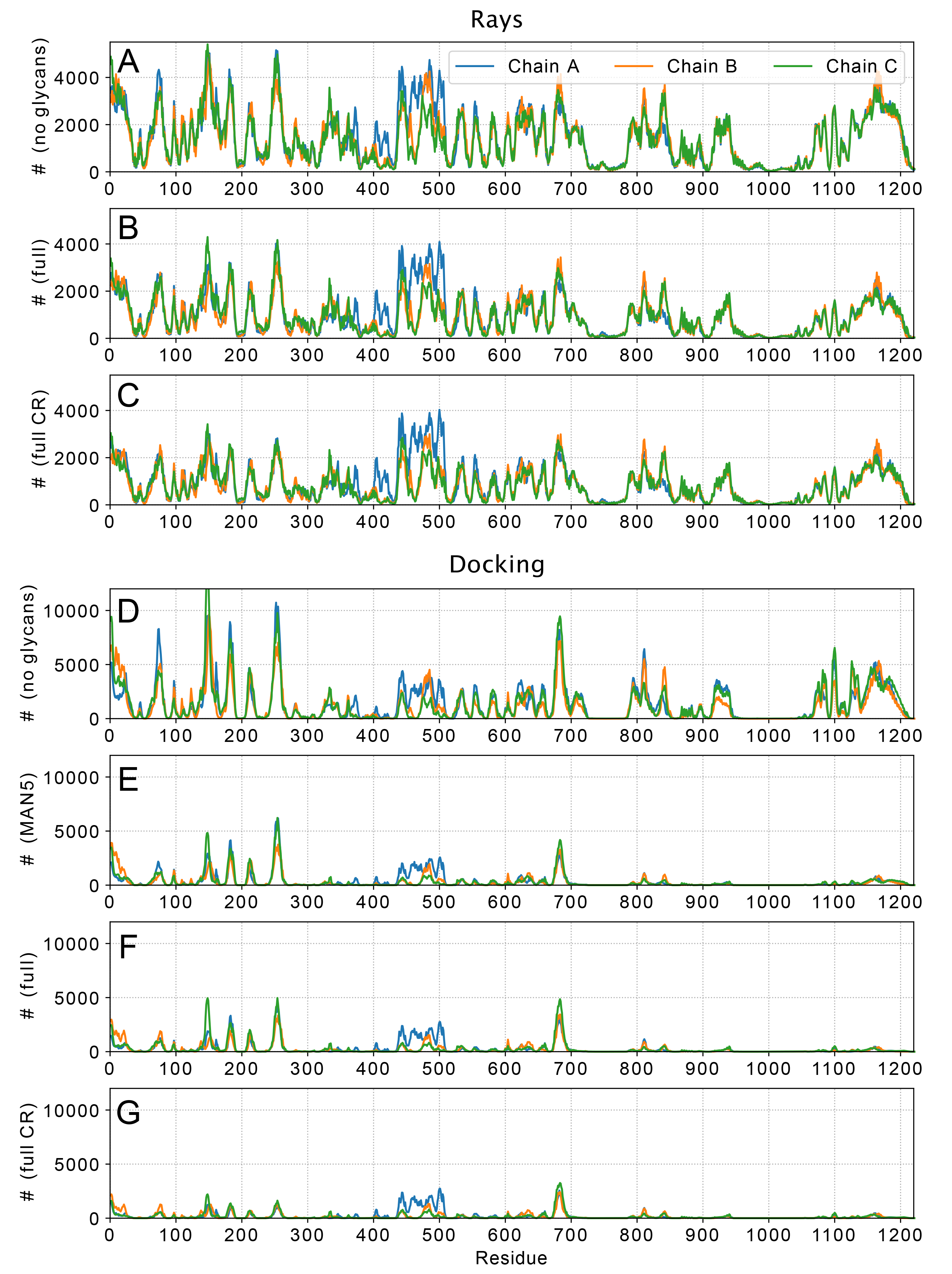

Supplement: S3 Fig — (A-C) Number of ray hits without glycans (“no glycans”), with full glycans (“full”, S1(B) Fig), and with full glycans and S protein crowding (“full CR”). (D-G) Monte Carlo rigid-body docking hits without glycans (“no glycans”), with Man5 glycans (“Man5”, S1(B) Fig) and with full glycans (“full”), as well as with full glycans and S protein crowding (“full CR”). (TIF) [file pcbi.1008790.s004.tif]

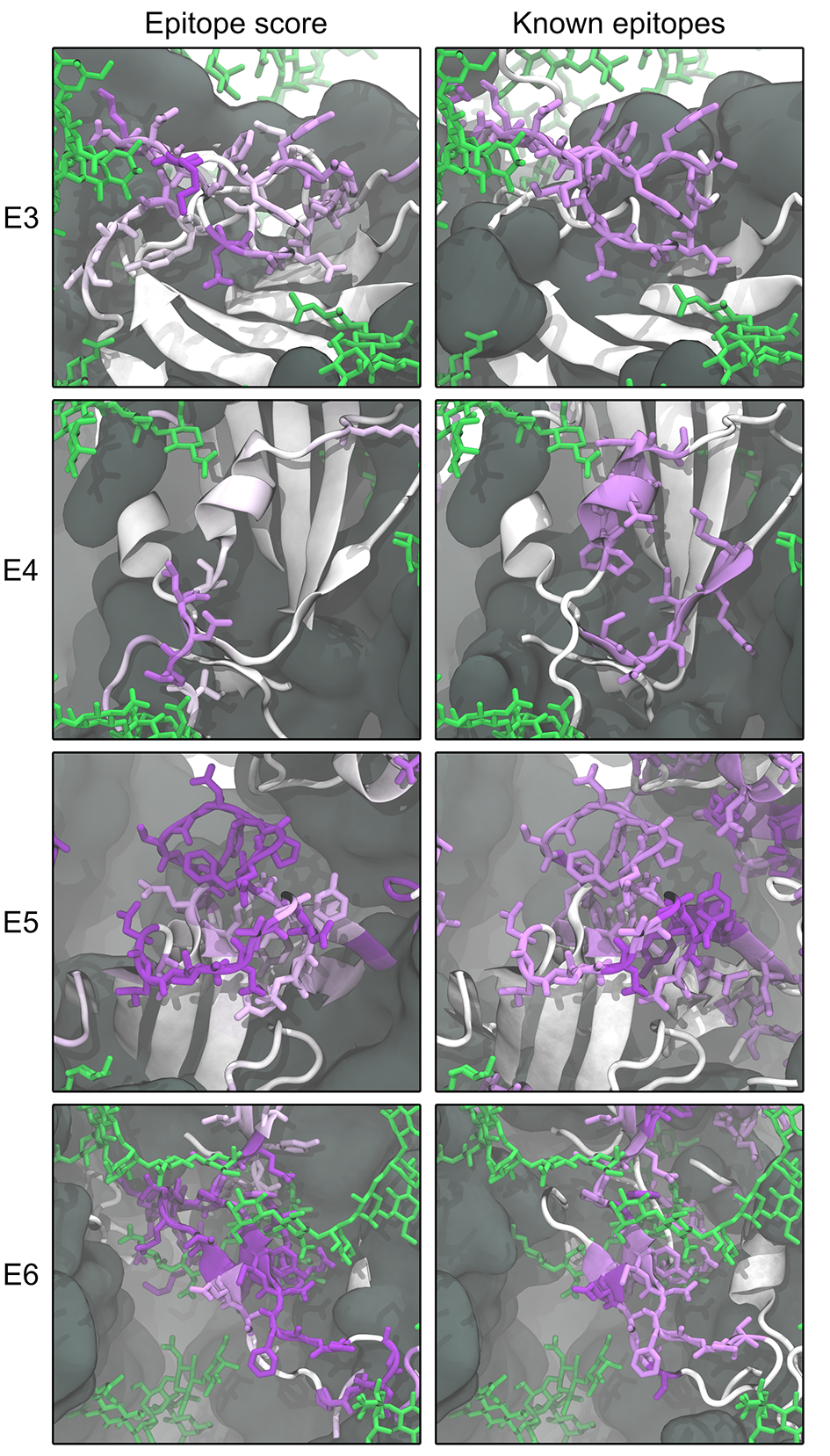

Supplement: S4 Fig — Glycans are shown in green licorice representation. Left panels: Epitope candidates shown in cartoon representation with purple color intensity indicating epitope consensus scores. Residues with epitope consensus score >0.1 are shown in licorice representation. Right panels: Epitopes described in previous works shown in cartoon and licorice representation, with higher purple color intensity indicating reported binding to multiple distinct antibodies. (TIF) [file pcbi.1008790.s005.tif]

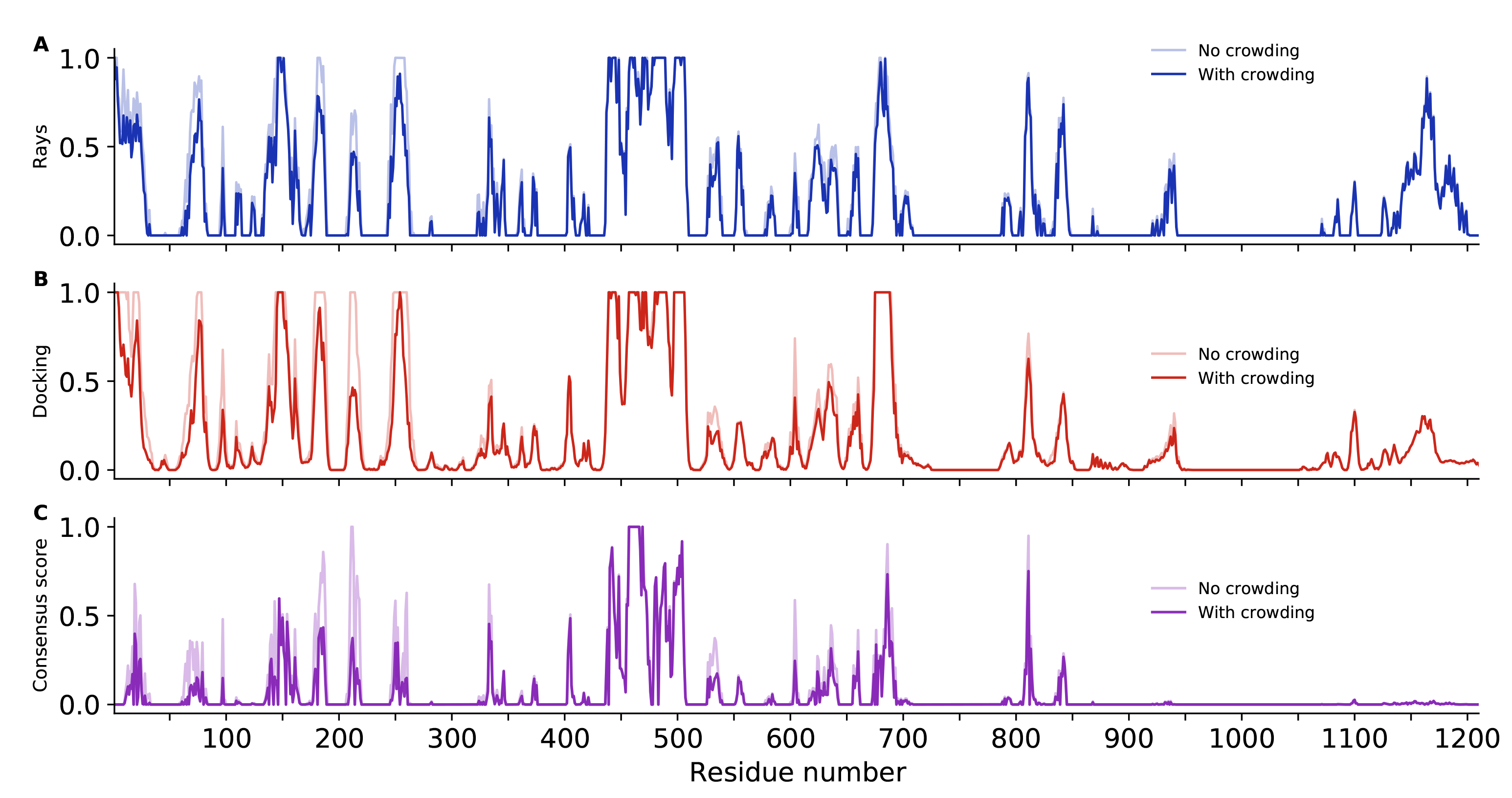

Supplement: S5 Fig — (A) Ray, (B) docking and (C) consensus scores with (thick line) and without crowding being taken into account. (TIF) [file pcbi.1008790.s006.tif]

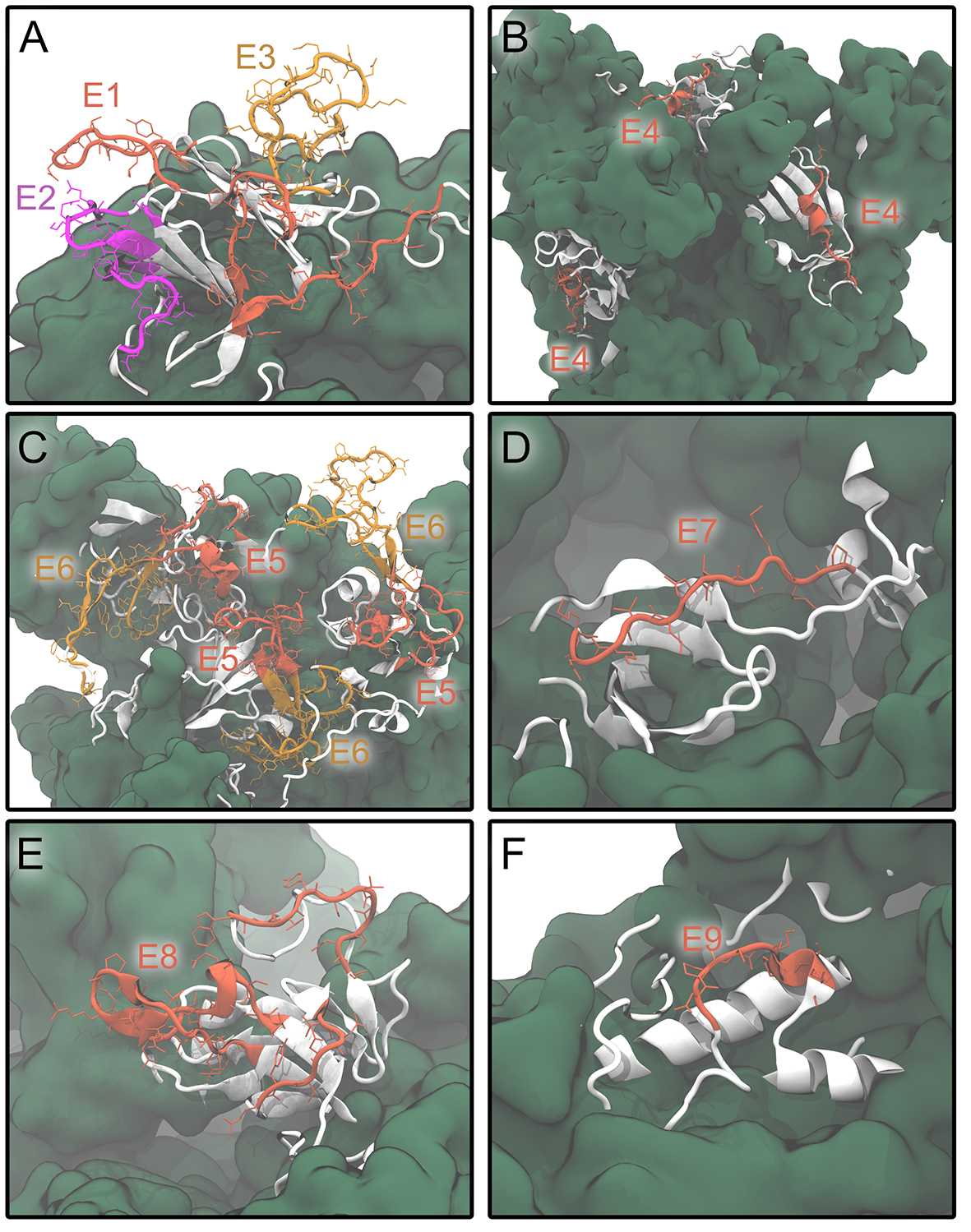

Supplement: S6 Fig — Epitope candidates are shown in red, orange and purple cartoon and licorice representation. Neighboring residues are shown in grey cartoon representation. (TIF) [file pcbi.1008790.s007.tif]

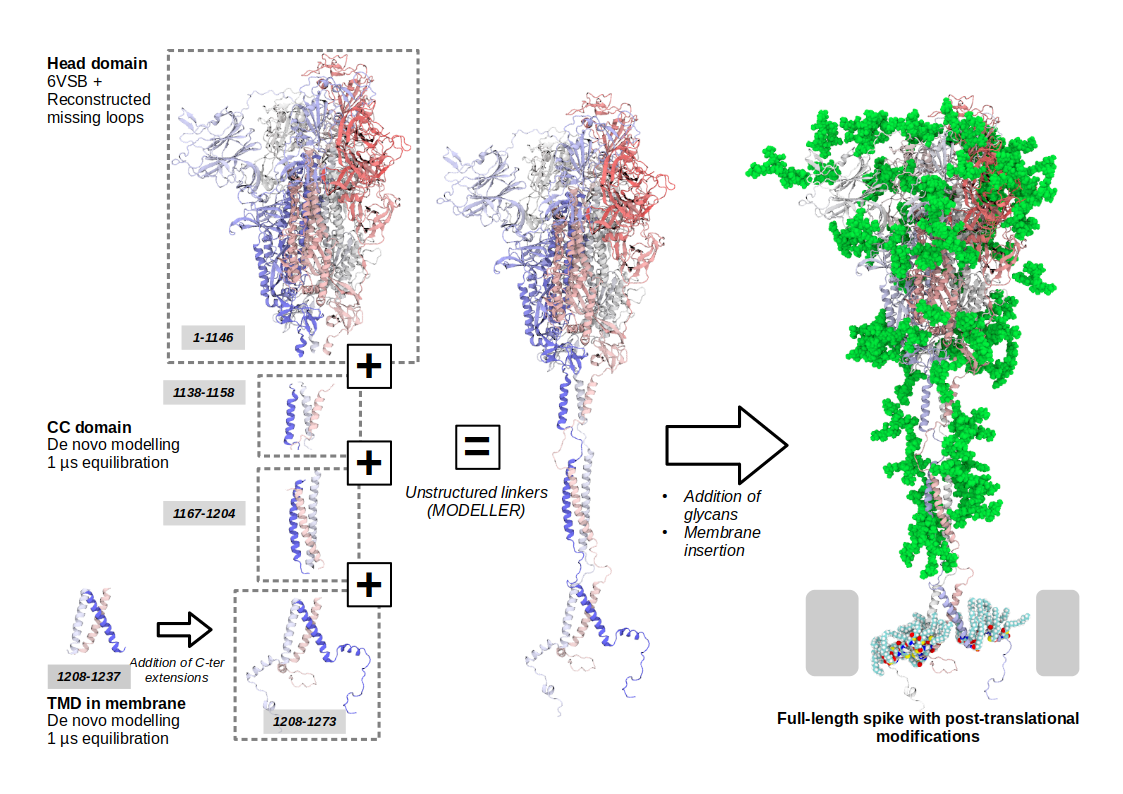

Supplement: S7 Fig — For clarity, we do not show the solvent and membrane. (TIF) [file pcbi.1008790.s008.tif]

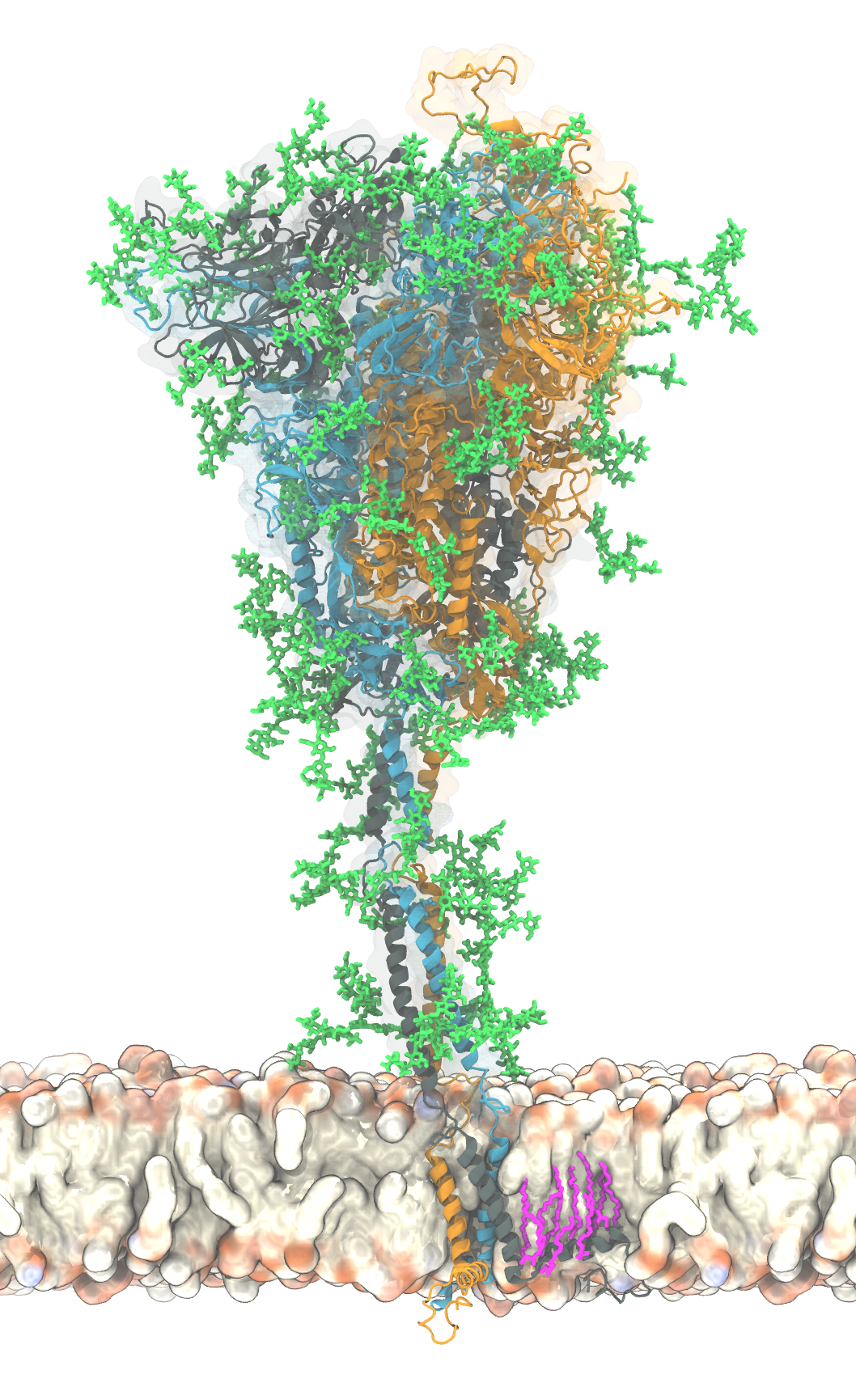

Supplement: S8 Fig — The chains are differentiated by color. Palmitoylated cysteine residues are shown in pink licorice (only one chain shown for clarity). Glycans are shown in green licorice representation. We show a section of the membrane to highlight the transmembrane domain of S. (TIF) [file pcbi.1008790.s009.tif]

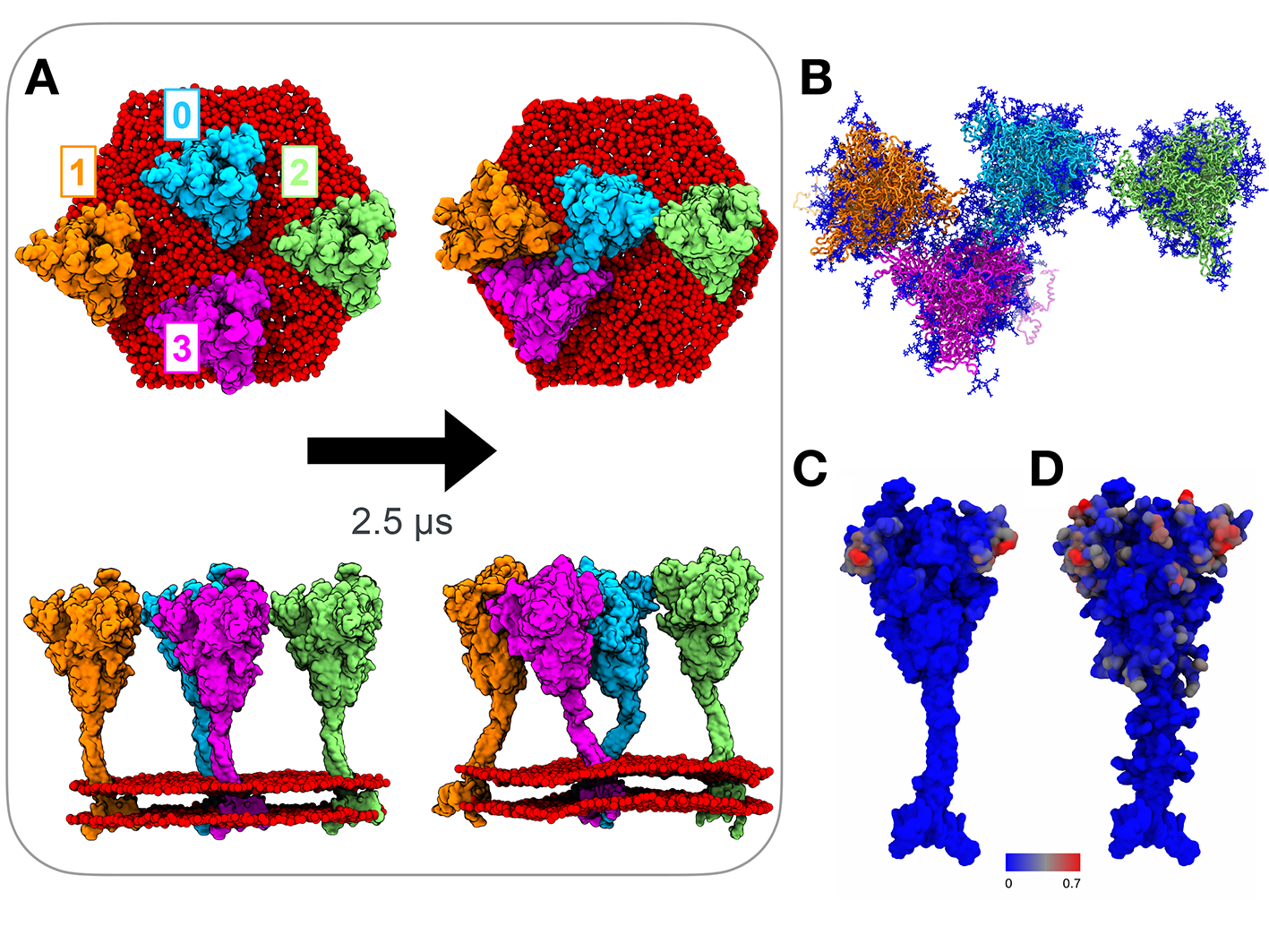

Supplement: S9 Fig — (A) Snapshots of the 4-spike system from above (top row) and in side-view (bottom row) at the beginning (left) and end (right) of the MD trajectory. While the transmembrane regions move relatively little, spike heads form spike-spike interactions because of significant bending at the “knee” (CC1—CC2 joint). These interactions persist on the simulation timescale. (B) Visualization of the glycans in the final configuration (blue sticks). Glycans mediate spike-spike contacts. (C and D) Maps of time-averaged spike-spike contact probability mediated by amino-acids (C) or amino-acids and glycans (D) from the MD trajectory (color bar: contact probability). Interactions are located exclusively on lateral faces of the spike head. (TIF) [file pcbi.1008790.s010.tif]

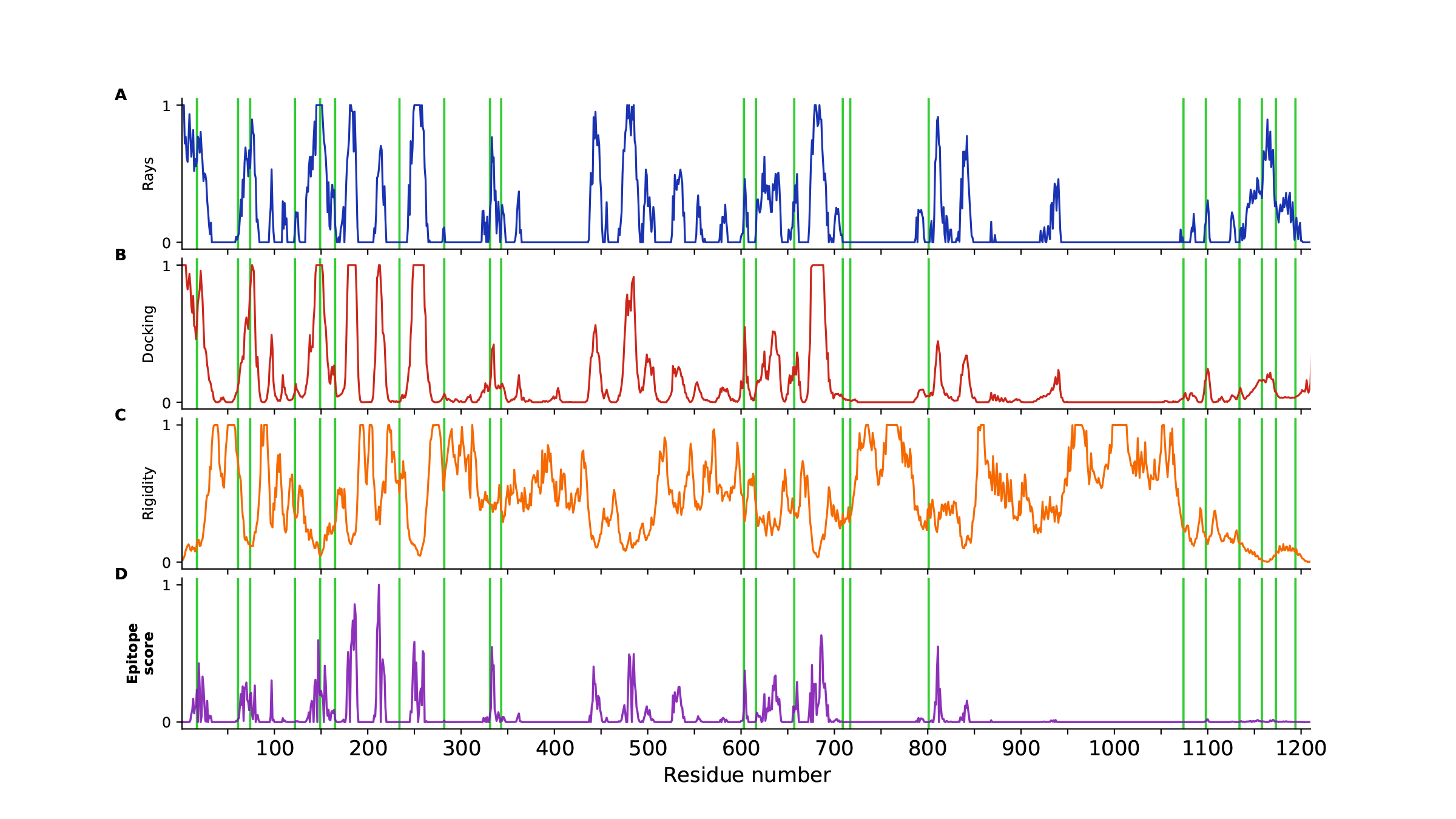

Supplement: S10 Fig — (A, B) Accessibility, (C) rigidity and (D) consensus score calculated taking only into account the chains with down RBDs. (TIF) [file pcbi.1008790.s011.tif]
