# Supplementary material for: Computational epitope map of SARS-CoV-2 spike protein
Source: PLoS Comput Biol. 2021 Apr 1;17(4):e1008790. doi: 10.1371/journal.pcbi.1008790 (PMC8016105; doi:10.1371/journal.pcbi.1008790)
Supplement: Attachment — Submitted filename: rebuttal.pdf [file pcbi.1008790.s013.pdf]

Response to Reviewer Comments

We thank the Editor for handling our manuscript, and thank the Reviewers for their careful assessment. In the following, we provide our answers to the Reviewers' remarks with the respective modifications to the manuscript. We are confident that our answers are convincing and that the outcome of this review significantly improved the quality of our manuscript.

Please note that the tiny changes in some of the reported numbers are the result of us now consistently analyzing the full 2.5 microseconds of MD simulations over all four spike proteins.

Below are the answers to the specific Reviewer's comments including the modifications of the manuscript text:

Reviewer 1

R1: "In this manuscript the author present further analysis of MD simulations recently published in Turoňová et al, Science (2020), doi: 10.1126/science.abd5223. This additional information concerns:
1) the ability of the S glycans to shield the spike protein from the immune system and
2) the prediction of epitopes that can be targeted for vaccine design and development
I find that such information is indeed important to share publicly, however I am not entirely convinced that it justifies a second publication based on the same set of experiments."

A: We completely agree with the Reviewer that sharing the description of our modelling work and the result of our analysis with the community is important. While our simulations were integral to interpret cryo-electron tomography data (Turoňová et al, Science (2020)), in this manuscript we not only report a completely independent and thorough analysis, but also a detailed description of our modelling choices and methods. The work published in Science represents a convincing validation of our model's accuracy, and allows us to focus here on illustrating that atomistic structural modelling and dynamics are useful tools to identify potential epitopes on viral proteins.

R1: "Further to this, I found aspects on the aforementioned analysis that I found should be re-examined. More specifically, in regard to part 1) the analysis of the accessible surface was done through rigid-body docking the CR3022 antibody Fab region and also through a process called "ray" where the protein was illuminated by diffuse light to identify areas of higher accessibility. The results obtained through there two methods are dramatically different, for instance the glycan shield determines an accessibility reduction of 40% (ray) and 87% (docking). This is a very large difference as a reduction of 40% still signifies wide accessibility, meanwhile a reduction of 87% dramatically precludes it. I

may have misunderstood this analysis, however the authors do not comment on such discrepancy."

A: We indeed used two alternative methods to quantify the accessibility to the surface of spike, which lead to different numerical results. We did expect such discrepancy. Whereas the ray analysis provides us with an upper bound to the accessibility, the rigid body docking gives us a lower bound, because it does not take into account any induced fit from the interactions between glycans and antibody, therefore underestimating accessibility. Importantly, the two methods are consistent in identifying regions of high and low accessibility (Fig. 4 A and B). Combining the two methods leads to a more accurate numerical estimate. We thank the Reviewer for giving us the opportunity to clarify our analysis choices and we adapted the manuscript accordingly in the Results section "Accessibility of the S ectodomain.":

We consider ray and docking analyses to be complementary: The ray analysis provides an upper bound to the accessibility, because the thin rays can penetrate more easily through the glycan shield than antibodies, whereas the rigid body docking gives a lower bound, because it does not take into account any induced fit from the interactions between glycans and antibody. Importantly, the two methods are consistent in identifying regions of high and low accessibility (Fig. 4 A and B).

R1: "In regards to part 2) the scoring function designed by the authors identifies a set of 9 epitopes that include 2 known ones. This point is highlighted as proof of the robustness of the score, yet those known epitopes are part of the glycan unshielded RBD and not so difficult to identify in general, as the RBD is the known target for the interaction with the ACE2 receptor and considering that the scoring function (rightfully) promotes unshielded regions. Notably, the score penalizes these very regions in terms of flexibility, which is an aspect that was not addressed in the manuscript. A proof of the robustness of this epitope scoring function would be in my opinion to test some of the unknown predicted epitopes experimentally."

A: We agree with the Reviewer that the epitope score must identify unshielded regions of the RBD, and that the identification of known epitopes is only a partial validation. However, in addition to correctly identifying the RBD as epitope candidate, our analysis predicted a non-RBD epitope that was later independently reported to be the binding site for an allosteric nanobody (Schoof et al., https://www.biorxiv.org/content/10.1101/2020.08.08.238469v2 , see also reply to Reviewer 3). Importantly, this information became available only after we had deposited our preprint on bioRxiv. We consider this independent verification a strong form of validation of our methodology. We agree with the Reviewer that further experimental tests of our predictions would be extremely interesting and we are in contact with experimental labs to move in that direction. However, this analysis is beyond the scope of the current work and we

would like to avoid further delay given the urgency of the topic.

R1: "As a last point, I am afraid that the 'trimming' of the glycans to account for immature glycosylation is fundamentally wrong for two reasons. The first reason is that ER glycans would be large oligomannose types, such as Man9 and Man8, which are processed down to Man5 in the Golgi by alpha mannosidases; the Man5 conversion into complex N-glycans is then initiated by GnT1 also in the Golgi. Mammalian cells don't have any paucimannose, which is more common in plants and insects. As for the second reason, the glycans 3D structure depends on their sequence and shorter versions do not have necessarily the same structure, similarly to how a protein region may not retain the same conformational propensity if trimmed down."

A: We are grateful to the Reviewer for this remark because it allows us to clarify our reasoning. We did not want to model immature glycans, which are not exposed to the immune system, but rather to quantify variations of the glycan coverage due to experimental uncertainties of the glycosylation pattern. To clarify this point, we repeated the docking accessibility analysis by considering a full Man5 type of glycosylation (Fig. S1 and S3). To address the second point and analyse only realistic conformers of glycans, we generated Man5 trajectories by resampling from all Man5 sites present in our simulations instead of the simple trimming approach. A comparison between the original and the Man5-type glycosylation reveals very minute differences: the relative accessibility reduction (see SI) is 72% with simple trimming to Man5 and 75% with resampling.

These very similar values suggest that the coverage is robust to realistic variations in the glycosylation pattern.

Reviewer 2

R2: "In this work, the authors present a massive, all-atom MD simulation of a patch of membrane of the SARS-CoV-2 virus with 4 spike proteins. Much of the analysis and so forth, as presented, is good. However I have some comments for the authors to consider.

My main comment is that the authors have a really unique opportunity to provide scientists with a view of the spike proteins in a very crowded environment. The spacing of the spike proteins – while this close range may exist in some rare instances – is quite different than the average structures, which place the spike proteins further apart.

That said, this crowded configuration does indeed exist and thus this work stands apart in its ability to inform others about what happens to the dynamics, the shielding, the flexible hinges, etc. No other work does this.

But what is written somehow doesn't capture the essence of this most intriguing aspect of the work. The authors only make a few very minor comments about this aspect. Yet, to me, as someone who also studies the spike protein –

I think it is of utmost importance to analyze and I think it is quite interesting and worthy to be published."

A: We thank the Reviewer for her encouraging words, and thank her for suggesting further interesting analyses.

R2: "In the introduction, the comment about the glycans playing a role and validated by experiment should be updated. The final published version of Casalino et al. provides experimental validation within the same paper."

A: We updated the manuscript to report the experimental validation:

Intriguingly, several groups have shown experimentally that glycans not only shield the S protein but also play a role in the infection mechanism. [Casalino et al., Henderson et al., Mehdipour and Hummer]

R2: "I would have liked to see more discussion of the crowdedness of the system, and a finer analysis of what that means. How many contacts are made between the spikes here, and are all those contacts predominantly glycan mediated? The authors present a short analysis of this at a very high level, on page 4, but a map of the residues themselves (contact footprint, perhaps?) would be useful. Were these direct contacts made during system construction or do they form over time? In their now iconic image presented here as figure 1, e.g., the stalks are quite bent – I've always wondered – did the stalks start out that way, or did they move to that conformation over time, and are they sort of stuck like that, or is this terribly sampling limited? The authors don't provide any such information."

A: We are grateful to the Reviewer for suggesting a number of interesting analyses. To address the Reviewer's query on the bending of the stalks in the simulation, we now report side-views and top-views of the system in the starting configuration and after 2.5 microseconds in Fig. S9A, which shows that S proteins bend and make contacts over time. As shown in Fig. S9B, the glycan shields are in close contact. The contact points are highlighted in Fig. S9C and D. We stress that crowded assemblies of S are not unusual even if spike is distributed randomly on the viral envelope (Fig. 5 of Turoňová et al.). These new findings are discussed in the new paragraph "Collective behavior of S" in the Results section of manuscript:

Collective behavior of S Despite remarkably random distribution of S at the surface of the virions, densely populated patches are not uncommon (cf. Fig 5G in Turoňová et al). Multiple S will likely come in contact if simultaneously bound to a single ACE2 receptor or when cross-linked by antibodies. Taking advantage of our simulation setup, we analyzed interactions between S. During

2.5 µs we observed a number of contacts involving both glycans and protein surfaces, which resulted in a partial jamming of three of the S in a characteristic triangular arrangement of S head domains and with the fourth S only weakly interacting with two of its neighbors (Fig. S9A). In the jammed state glycans formed an extensive network of interactions, as could be seen in Fig. S9B. To quantify relative roles of particular residues and glycan moieties we computed a contact map of inter-S interactions (Fig. S9C and D). We found the majority of protein-mediated contacts to reside in the unstructured loops within NTD regions. Glycan-mediated contacts concentrate in the sequons located in the NTD and RBD areas and at the bottom of the S head. Despite their size, glycans located on the stalk were not involved in the inter-S contacts. Instead, they remained relatively shielded by the much more spacious head domains.

R2:"I also think the authors MUST deposit the full system models (PDB minimally, preferably actual simulation input files) as part of this work. There are likely other useful choices in terms of the many variable parameters, that the authors work will provide others, as we seek to understand things about this system."

A: We agree with the Reviewer. Following the journal's guidelines, we had uploaded already at submission time structure and topology files to a repository accessible to the Reviewers (https://doi.org/10.5281/zenodo.3906318). These files are now publicly available at https://doi.org/10.5281/zenodo.4442942 . We are also happy to find further ways of sharing our model.

Reviewer 3

R3: "This manuscript introduces a very interesting and a general approach to design novel antibodies to fight viral infections, including SARS-COV2. The approach is very elegant and based on combining multi-microsecond long molecular dynamics simulations of the target proteins in their native environment with bioinformatics-based analyses, which results in antibody-binding scores. Overall, the results in the manuscript are convincing and support the message of the authors. The manuscript is generally well-written and clear. It addresses a timely problem and should be of interest to PLOS Computational Biology readership and community in-general. Thus, I would be in favor of accepting the manuscript for publication in PLOS Computational Biology. However, I have some concerns that the author needs to address before it can be accepted."

A: We thank the Reviewer for this positive assessment.

R3: "1. Due to the plethora of on-going efforts to simulate full-length structure of SARS-COV2, including some of the recent work from these authors and Amaro lab, it is important to have a consensus on the full-length structure of

SARS-COV2. Therefore, authors should discuss about their modelling approach in the light of recently published work by Amaro lab (ACS Central Science, 2020, 6, 10, 1722-1734)."

A: We agree with the Reviewer that it would be desirable to have a consensus atomistic structure of the full-length S protein. However, due to experimental uncertainties in parts of the protein structure, we believe that it is actually good to have different modelling approaches to cover more of possible reconstruction solutions. Overall, the modelling decisions in Casalino et al. (ACS Central Science, 2020, 6, 10, 1722-1734) are very similar to the ones taken in this work, with some differences: 1. Casalino et al. simulated the spike both in the "one-open" and in the fully closed conformation, whereas here we focused on the "one-open" structure. 2. There are some differences in how we modelled post-translational modifications including glycosylation. We stress that these choices are unlikely to dramatically affect the representation of the spike head, which emerges as the most relevant for our study. We added the following detailed discussion of all differences to the Supplementary Information of the manuscript:

Comparison with the model by Casalino et al Our modelling decisions are similar to those by Casalino et al. except on the following points:

1. We modelled the spike head with one chain open and two chains closed (6VSB cryo-EM structure). In addition to this configuration, Casalino et al. also considered a spike model with all three chains closed (6VXX cryo-EM structure).

2. We manually modelled the C-terminal domain as an alpha-helix flanked by disordered linkers, which we relaxed by MD. By contrast, Casalino et al. used the I-TASSER structural prediction server. Interestingly, they retained a model with an alpha-helix similar to ours.

3. We decided to palmitoylate all cysteines in the TMD and C-terminal domains. Casalino et al. palmitoylated only cysteines 1240 and 1241.

4. Similar to Casalino et al., we followed the N-glycosylation determined by Wanatabe et al., with essentially the same glycan types (including large tetra-antennary glycans on the stalk) and small differences in the details of sialylation and/or fucosylation. However, we did not include any O-glycans. In addition, Casalino et al. considered a system with N165 and N234 mutated to alanine. We instead performed a large-scale resampling of the simulations to understand the role of glycan size and composition on shielding.

Overall, most differences relate to secondary structure assignment in the stalk and global structural modelling of the TMD/C-terminal region including the palmitoylation pattern. As high-resolution structural data of these regions

are currently lacking, the disparity of modelling decisions between independent studies may actually be useful in exploring plausible solutions. Also, we note that possible modelling errors of the TMD/C-terminal regions are expected to have very little bearing on the protruding, solvent-exposed regions of the spike which are most relevant for the present epitope search.

R3: "2. Authors mentioned that during the MD simulations, S protein dynamically interacted with its neighboring copies. Does S protein forms stable interactions with the neighboring copies? Authors should provide a detailed analysis, maybe using clustering-based methods, to highlight the dynamic interactions between S proteins."

A: We thank the Reviewer for raising these interesting and important points. We now provide a detailed analysis of the interactions between neighboring S proteins. Top-views and side-views of the system at initiation and after 2.5 microseconds are presented in Fig. S9, showing how the S proteins interact with each other. We also provide a map of direct protein-protein contacts and of contacts mediated by glycans in Fig. S9. We observe that the S proteins readily interact with each others both directly and mediated by glycans, predominantly at the NTD of the S protein. Due to the sampling limitations these interactions persist over the course of the simulation. These new findings are discussed in the new paragraph "Collective behavior of S" in the manuscript (see Reply to Reviewer 2 above).

R3: "3. In order to perform bioinformatics-based epitope scoring, authors selected 220 x 4 snapshots from their 2.2us-long MD simulations. How different were these snapshots? What was the criterion to select snapshots at 10ns intervals? This information will help readers not only to reproduce the data but also help them to intelligently apply this method to other important systems as well."

A: We leveraged the "enhanced sampling" strategy from having four spikes in the simulation box, allowing us to analyze four times as many configurations per simulation frame. We believe that this is a useful but not required strategy to apply the method to other important systems as well. The docking strategy using coarse-grained Monte Carlo simulations of an antibody and the spike protein were performed for all spikes and 250 frames. Because this is a computationally demanding task – with a total of 200 million individual displacements (or "sweeps") – we chose a sampling frequency of 10 ns as the maximal frequency which was manageable in the frame of this work. As a bonus, this sampling frequency should ensure uncorrelated configurations of the glycan chains. We believe that this is a good compromise approach also for the investigation of other systems.

R3: "4. In-order to be exhaustive in their approach (which I really liked),

authors performed epitope scoring under different glycan conditions. However, it seems like this analysis is based on the assumption that S protein dynamics will not be altered under different types of glycans. I think this is a strong assumption to make and authors should properly justify it."

A: We agree with the Reviewer that glycans not only impact accessibility, but could also impact the spike's dynamic. Assessing this impact requires comparing the dynamics between fully glycosylated and unglycosylated spikes. However, in this manuscript we focused on simulating only the natively glycosylated spike. Also, we do not think that different glycan coverages would dramatically alter the dynamic of spike. As can be appreciated directly from visual inspection of the MD trajectories (Movie S1), the dynamics of the glycans is much faster and uncoupled with respect to the spike's dynamics.

R3: "5. Based on the starting S protein structure, 2 RBDs exists in down conformation and 1 RBD is in up conformation. Did authors captured conformation-dependent epitope scores for the regions residing on RBDs? Authors should include a discussion and epitope scoring data on this as well."

A: The Reviewer raises a very interesting point, and it is likely that some epitopes get exposed during spike's conformational changes. Unfortunately, we could not sample any major conformational change in our simulations. However, we can estimate what the epitope score would look like if all RBDs were in a fully "open" or fully "closed" conformation. In our analysis, we consider each chain of spike separately. Epitope scores in new Fig. S10 report separately on the closed conformations.

A comparison with Fig. 4 highlights that the only sizeable differences in accessibility occur in the RBD region. Here, the two protein chains in closed conformation show consistent scores, suggesting that the scores are general for open and closed conformations, respectively. We now comment on this useful piece of analysis in the Results section of the manuscript:

"In our simulation, two of the RBD domains were sampled in closed conformation and one in open conformation. To assess the effect of open and closed states, we computed the accessibility and consensus scores for all eight protein chains with closed RBD conformation (Fig. S10). A comparison with Fig4 highlights that the only sizeable differences in accessibility occur in the RBD region."

R3: 1. Authors state that "On SARS-CoV-2 virions, S proteins occasionally form dense clusters, which may enhance the avidity of the interactions with human host cells [23]". However, according to reference 23 (Figure 2A), there is no significant tendency to cluster. This point should be addressed.

A: Indeed, the reference shows a random distribution of the S proteins on the virions. In fact, this random distribution implies that the spikes are not spaced

equidistantly on the surface, but that instead regions of lower and higher spike density occur. Figure 5G in the same reference highlights such a region of high S density. Therefore, even without a pronounced tendency of the spike proteins to form clusters, nonspecific cluster formation will occur on most virions.

R3: 2. Based on my understanding, epitope 5-6 seems to be in good correspondence with the recent pre-print (https://www.biorxiv.org/content/10.1101/2020.08.08.238469v2) on nanobody design. Authors should include a discussion of their results with this CryoEM study. Interestingly, epitope 2 seems to be in good correspondence with the allosteric nanobody reported in the above pre-print.

A: We thank the Reviewer for pointing out this exciting study. The residues involved in binding the allosteric nanobody have been added on Figure 4 and a reference to this paper has been included in our listing of RBD-targeting antibodies. In addition, we now discuss that E2 is consistent with the allosteric nanobody binding site in the Discussion section:

Interestingly, epitope E2 includes residue 207 and is in close proximity to residue 177, both of which have been reported by Schoof and co-workers [Schoof et al.] to be involved in binding an allosteric nanobody . We propose that E2 could represent the full binding site of this nanobody, which has not been mapped completely. Thus, our method may also be used to complement experimental characterizations of epitopes.

R3: 3. In the methods section. authors state "the initial distance between the center of mass of the stalks if neighboring S was about 15nm." This does not guarantee that ectodomain, to be specific RBDs are not interacting in the initial setup, thus making the system biased. Authors should indicate minimum distance between S proteins, in their initial setup.

A: To clarify this point, we now include a top-view and side-view of the initial system setup and of the system after 2.5 microseconds of the simulation in **Fig. S9**. The manuscript text now addresses this issue and reads: we set the initial distance between centers of mass of the stalks of neighboring S to about 15 nm. This guaranteed at least 1 nm of spacing between two S's most extended glycans and thus no contacts between S in the initial configuration (cf. Fig.S9A).

R3: 4. Figure 4 is difficult to interpret and is too crowded, authors should make it more reader friendly.

A: We agree that the original version of Figure 4 was difficult to interpret. We have revised the Figure by removing redundant known epitopes, expanding the consensus score row, generally decluttering the layout and adding a separate bar highlighting residues involved in known epitopes. We think that these mod-

ifications make the Figure more accessible.The caption has also been modified:

"Black rectangles show known antibody binding sites, also indicated in black along the S sequence in the bottom box."

R3: 5. Reference 30 in Supplementary Methods doesn't seem to be on monte-carlo based rigid body docking. This should be corrected.

A: We acknowledge that the phrasing of the sentence caused confusion. Indeed, the reference does not discuss docking simulations, but we used the rigid-body Monte Carlo simulation framework presented therein for our high-temperature rigid docking simulations. The revised sentence now reads "The Fab of antibody CR3022 (PDB ID: 6W41 [Yuan et al.] was used for a coarse-grained rigid body Monte Carlo (MC) docking analysis according to the simulation procedure described in [Kim & Hummer]."
